# Magnesium Chloride promotes Osteogenesis through Notch signaling activation and expansion of Mesenchymal Stem Cells

**DOI:** 10.1038/s41598-017-08379-y

**Published:** 2017-08-10

**Authors:** Juan M. Díaz-Tocados, Carmen Herencia, Julio M. Martínez-Moreno, Addy Montes de Oca, Maria E. Rodríguez-Ortiz, Noemi Vergara, Alfonso Blanco, Sonja Steppan, Yolanda Almadén, Mariano Rodríguez, Juan R. Muñoz-Castañeda

**Affiliations:** 1 Maimonides Institute for Biomedical Research (IMIBIC), 14004 Cordoba, Spain; 2 Nephrology Service, Reina Sofia University Hospital, 14004 Cordoba, Spain; 3 University of Cordoba, 14004 Cordoba, Spain; 4 Spanish Renal Research Network (REDinREN), Institute of Health Carlos III, 28029 Madrid, Spain; 5 Laboratory of Nephrology, Health Research Institute-Jiménez Diaz Foundation, 28220 Madrid, Spain; 6 Dept. of Anatomy and Comparative Pathology and Anatomy, University of Cordoba, 14014 Cordoba, Spain; 7 Fresenius Medical Care Deutschland GmbH, 61352 Bad Homburg, Germany; 8 Internal Medicine Service, Reina Sofia University Hospital, 14004 Cordoba, Spain; 9 Biomedical Research Center Network on Physiopathology of Obesity and Nutrition (CIBERobn), Institute of Health Carlos III, 28029 Madrid, Spain

## Abstract

Mesenchymal stem cells (MSC) are osteoblasts progenitors and a variety of studies suggest that they may play an important role for the health in the field of bone regeneration. Magnesium supplementation is gaining importance as adjuvant treatment to improve osteogenesis, although the mechanisms involving this process are not well understood. The objective of this study was to investigate the effects of magnesium on MSC differentiation. Here we show that in rat bone marrow MSC, magnesium chloride increases MSC proliferation in a dose-dependent manner promoting osteogenic differentiation and mineralization. These effects are reduced by 2-APB administration, an inhibitor of magnesium channel TRPM7. Of note, magnesium supplementation did not increase the canonical Wnt/β-catenin pathway, although it promoted the activation of Notch1 signaling, which was also decreased by addition of 2-APB. Electron microscopy showed higher proliferation, organization and maturation of osteoblasts in bone decellularized scaffolds after magnesium addition. In summary, our results demonstrate that magnesium chloride enhances MSC proliferation by Notch1 signaling activation and induces osteogenic differentiation, shedding light on the understanding of the role of magnesium during bone regeneration.

## Introduction

Bone growth is a process required in a wide variety of conditions in which functional restoration of damaged bone is needed. There are pathological conditions such as polytraumatism, bone tumors, degenerative diseases, orthopedic surgeries, osteonecrosis, osteotomy or non-union fractures where a combination of prosthesis implantation and enhanced osteogenesis is necessary^1, 2^. The standard treatment in some of these conditions require autologous or allogenic cancellous bone transplantation, supplemented with growth factors and/or progenitor cells. These alternatives have limitations such as surgical complications, elevated cost and/or immunogenic rejection^3–5^. Therefore, the search for new strategies to improve bone grafts has risen widely over the last years. An optimal biomaterial should be simple, biologically safe, biocompatible and with a high degree of interaction with the patient’s bone tissue to favor proliferation and differentiation of progenitor cells into an osteogenic phenotype.

Nowadays, there are many types of scaffolds such as calcium phosphate-based materials (extensively studied as bone scaffold for tissue engineering), polymeric (collagen, fibrin, alginate, silk, hyaluronic acid, or chitosan), composite (two or more distinct materials), metallic (magnesium or titanium) or third generation scaffolds that combine the aforementioned materials with stem cells, growth factors, cytokines, etc^1^.

Magnesium (Mg^2+^) is particularly interesting because of its abundance in the organism, where it participates in numerous biological processes, like osteogenesis of progenitor cells. In addition, low Mg^2+^ concentrations have been associated with osteoporosis or osteopenia^6^. In biomaterial engineering, Mg-based implants have been used to enhance bone formation “*in vivo*”^7–10^. Due to corrosion resistance of Mg^2+^ alloys^11^, new formulas based on Mg^2+^ are being investigated. Currently, Mg^2+^ is used in combination with other materials as calcium and phosphate, or as Mg-coated structures to enhance osteogenesis. However, little is known about the mechanisms whereby Mg^2+^ salts affect osteogenesis and bone formation. It is known that Wnt/β-pathway and Notch signaling are involved in bone marrow mesenchymal stem cells (MSC) osteogenesis^12, 13^. Although other works show that Notch signaling activation is also related to the maintenance of stemness^14^ even with the inhibition of osteogenesis^15^.

The characterization of the pro-osteogenic effects of Mg^2+^ will add knowledge on the biology of bone cells; thus, new or improved strategies can be developed aiming to enhance osteogenesis, and osseointegration of bone prosthesis.

The present study evaluates the effects of magnesium chloride on osteogenesis of bone marrow MSC and its capability to repopulate decellularized bone scaffolds. Moreover, the mechanisms whereby magnesium chloride triggers its pro-osteogenic effect are also investigated.

## Results

### Moderately high concentrations of Mg^2+^ increase osteogenesis and mineralization of rat MSC

**Figure 1:**
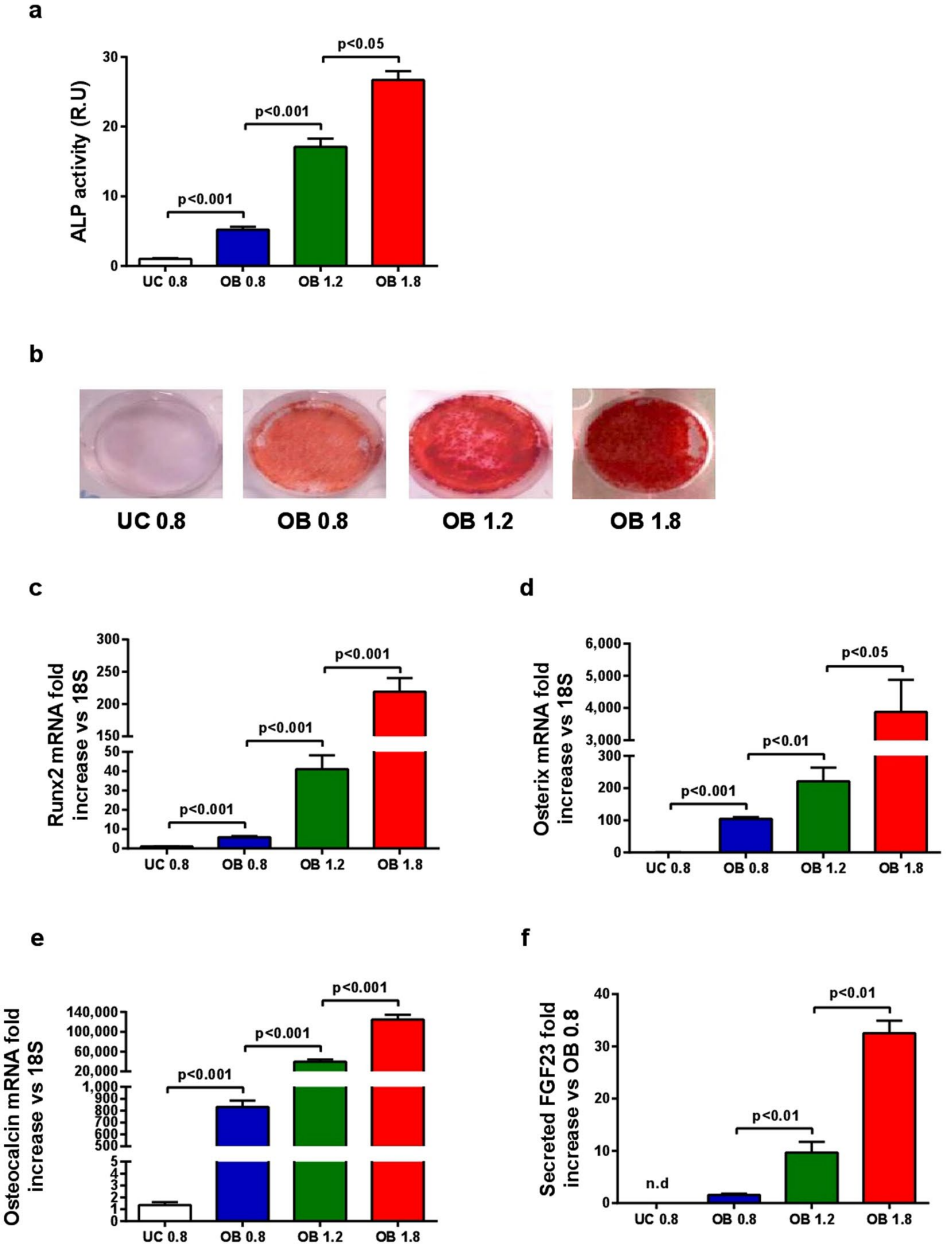
Effects of MgCl_2_ supplementation on osteogenesis and mineralization of rat MSC. (**a**) ALP activity was significantly and dose-dependently increased by Mg^2+^ concentration. (**b**) Matrix mineralization verified by Alizarin Red S staining was higher according to Mg^2+^ levels. (**c**) Osteogenic marker genes Runx2, (**d**) Osterix and (**e**) Osteocalcin expression was up-regulated according to the increase in Mg^2+^ concentrations. (**f**) Intact FGF23 in the liquid supernatant was increased according to Mg^2+^ concentrations. FGF23 was not detected in liquid supernatant of UC. UC - undifferentiated cells, OB - osteoblasts. Bars show mean ± SEM. n = 4.

Increasing Mg^2+^ concentrations (0.8, 1.2, and 1.8 mM) enhanced MSC osteogenesis (Fig. 1). In MSC differentiated into osteoblasts with 1.2 mM of MgCl_2_, alkaline phosphatase activity (ALP) increased by 4.2-fold (vs 0.8 mM) and by 6-fold with 1.8 mM of MgCl_2_, (Fig. 1a). Likewise, matrix mineralization, verified by Alizarin Red S, was more abundant in cells cultured with high Mg^2+^ concentrations (Fig. 1b).

The enhancement of mineralization was accompanied by a significant increase in the expression of osteogenic master genes such as *RUNX2* (Fig. 1c), regulator of the early osteoblast differentiation, *OSTERIX* (Fig. 1d), transcription factor required for the transition of pre-osteoblasts to osteoblasts, and *OSTEOCALCIN* (Fig. 1e), produced by mature osteoblasts. The addition of MgCl_2_ increased the expression of these genes in a concentration-dependent manner. As FGF23 is released by mature osteoblasts and osteocytes, the amount of FGF23 in the supernatant demonstrates the presence of mature osteoblasts. FGF23 production during 24 h was measured at 21 days of osteogenic differentiation. Undifferentiated cells did not produce FGF23 while in MSC differentiated into osteoblasts with 1.8 mM of MgCl_2_ FGF23 levels were 31-fold increased as compared with 0.8 mM (Fig. 1f).

**Figure 2:**
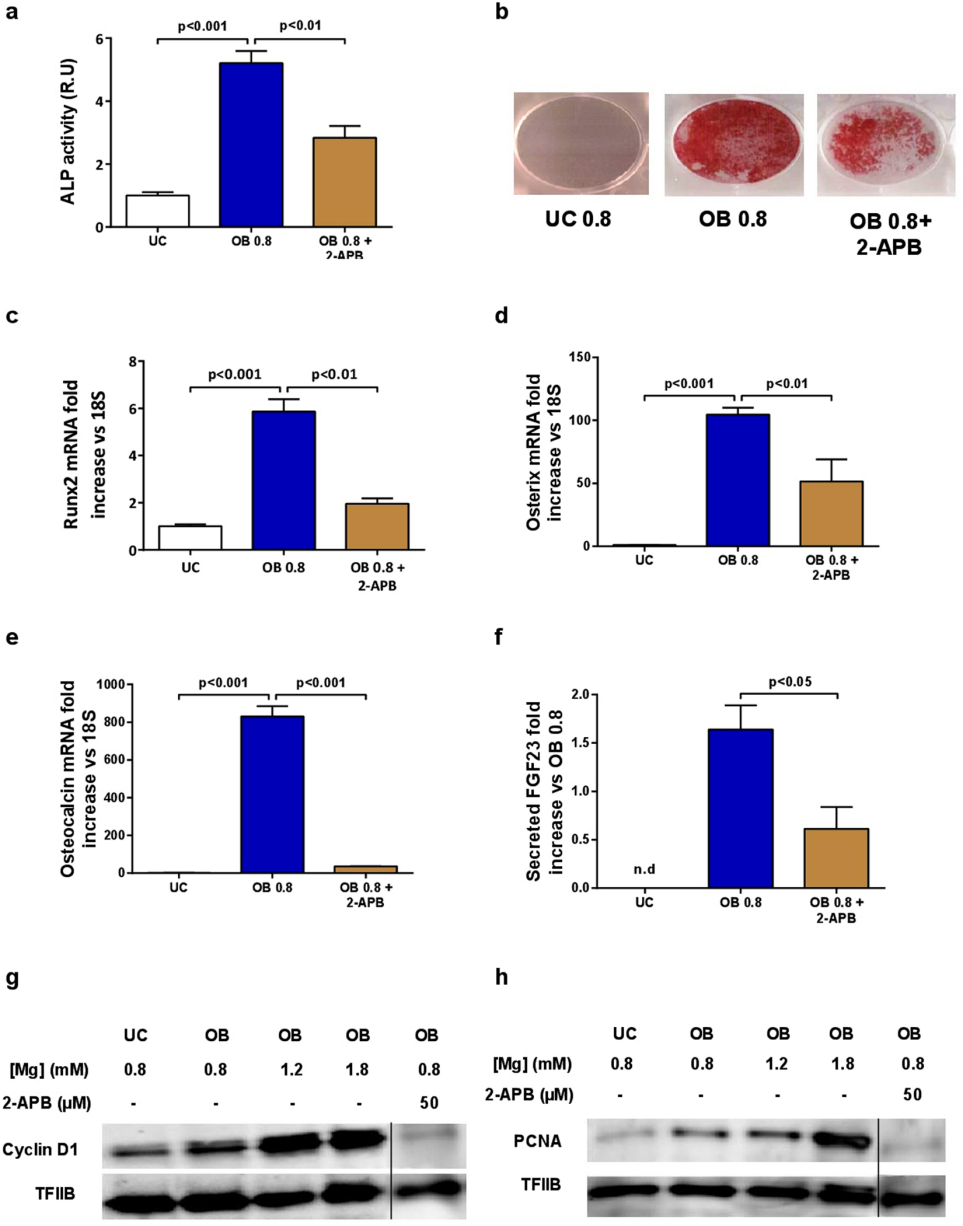
Decrease in intracellular Mg^2+^ reduces osteogenesis, mineralization and proliferation during differentiation of rat MSC into osteoblasts. Inhibition of Mg^2+^ entry by blocking TRPM7 with 2-APB (50 µM) significantly decreased (**a**) ALP activity and (**b**) matrix mineralization as assessed by Alizarin Red S staining. Specific osteogenic marker genes (**c**) *RUNX2*, (**d**) *OSTERIX* and (**e**) *OSTEOCALCIN* were down-regulated by inhibition of Mg^2+^ entry with 2-APB. (**f**) FGF23 production was decreased by inhibition of the Mg^2+^ transporter TRPM7 with 2-APB. (**g**) Western blots show an increased stimulation of Cyclin D1 and (**h**) PCNA according to Mg^2+^ concentrations at 21 days of osteogenic differentiation. TFIIB was used as a loading control, UC - undifferentiated cells, OB - osteoblasts. Bars show mean ± SEM. n = 4. Vertical black line separates results from different gels using the same exposure and protein load (see Supplementary Figures S3–S4).

The inhibition of the Mg^2+^ transporter TRPM7 by 2-APB produced a decrease in ALP activity (Fig. 2a) as well as in matrix calcification (Fig. 2b). It also resulted in a significant down-regulation of the osteogenic marker genes *RUNX2*, *OSTERIX* and *OSTEOCALCIN* (Fig. 2c–e, respectively). Moreover, 2-APB treatment reduced FGF23 secretion (Fig. 2f).

Protein levels of Cyclin D1 and PCNA, markers of cellular proliferation, were analyzed by Western blot. The expression of both proteins was increased in osteoblasts as compared with undifferentiated controls. The addition of Mg^2+^ to the medium induced a dose dependent increase in both PCNA and Cyclin D1 (Fig. 2g and h). Notably, the inhibition of the Mg^2+^ channel TRPM7 with 2-APB significantly reduced Cyclin D1 and PCNA protein expression.

### Magnesium increases osteogenic differentiation through activation of Notch1 signaling in MSC

**Figure 3:**
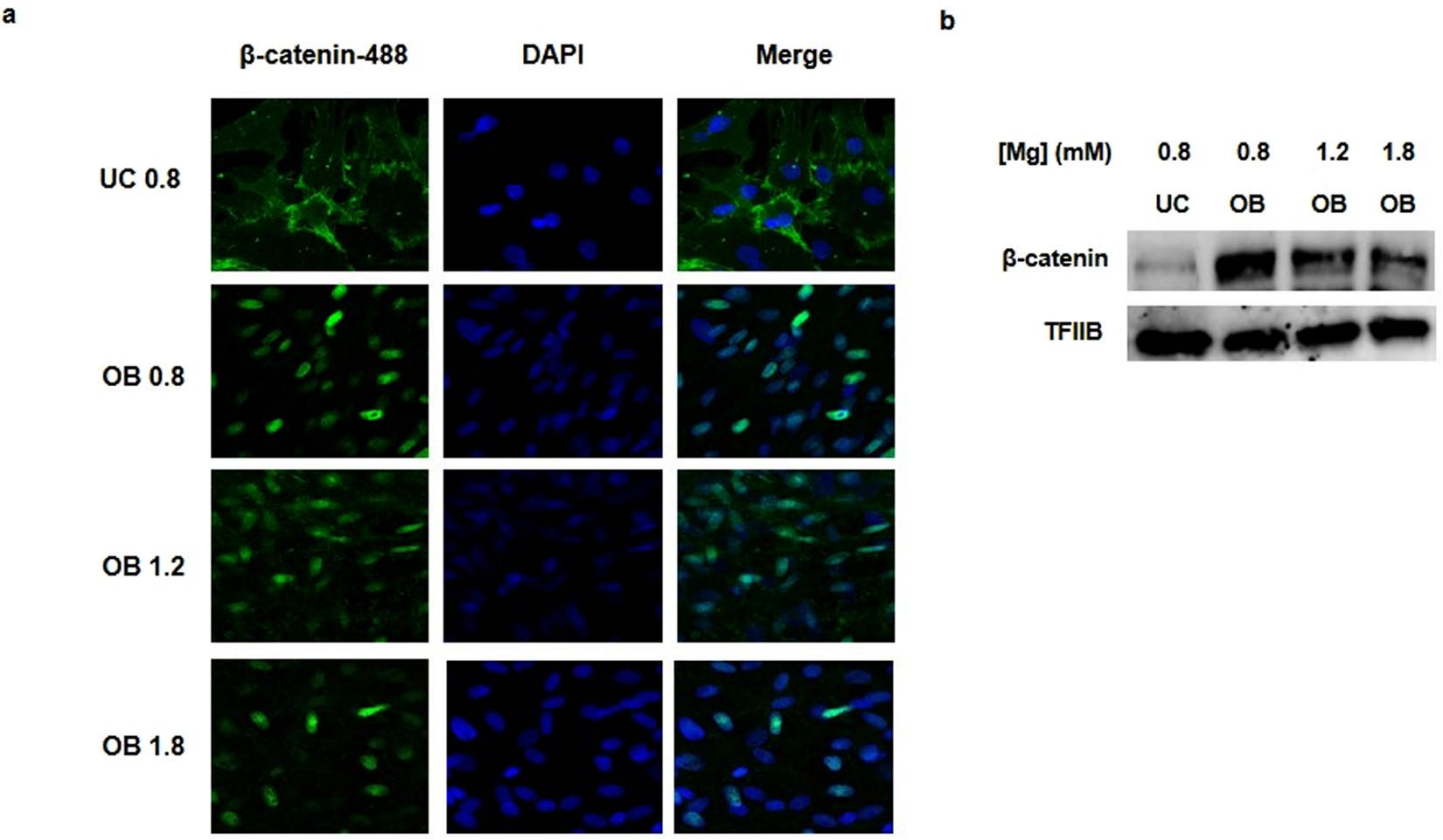
Mg^2+^ supplementation does not induce nuclear translocation of β-catenin. (**a**) Confocal microscopy showed translocation of β-catenin (green) to the nuclei in rat MSC cultured in osteogenic medium, but no significant differences were observed in groups with higher Mg^2+^ levels. (**b**) Western blot analysis of β-catenin for nuclear protein extracts. TFIIB was used as loading control. UC - undifferentiated cells, OB - osteoblasts. Original magnification: 400x.

The Wnt/β-catenin and Notch signaling are pathways closely involved in bone development. The effect of Mg^2+^ on both pathways was explored. Immunofluorescence analyses revealed that osteogenic differentiation is associated with nuclear translocation of β-catenin. However, increasing MgCl_2_ concentrations in the culture medium did not produce a further increase the nuclear translocation of β-catenin (Fig. 3a and b).

**Figure 4:**
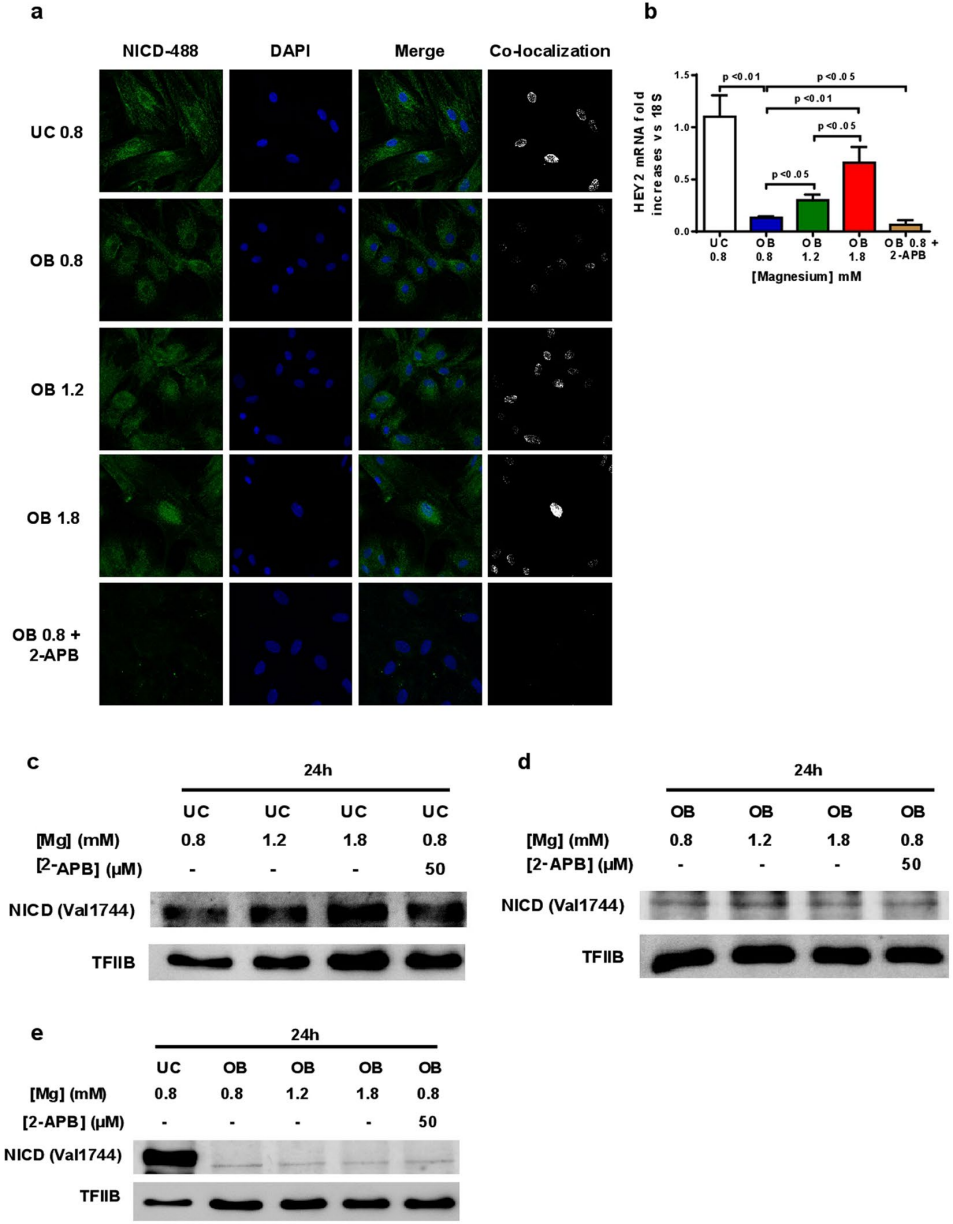
Moderately high Mg^2+^ levels induce Notch signaling activation. (**a**) Confocal microscopy of NICD protein in undifferentiated MSC (UC 0.8 mM), differentiated osteoblasts with basal levels of Mg (OB 0.8) or plus Mg supplementation at 1.2 mM (OB 1.2) or 1.8 mM (OB 1.8 mM) and osteoblasts with Mg channel block (OB 0.8 + 2-APB). First column represents NICD immunostaining in green color; second column corresponds with nuclei staining with DAPI; the third column is a merge composition of green and blue staining while the last column shows green pixels that matches with blue pixels. (**b**) Gene expression of the Notch target gene HEY2. (**c**) Western blot for nuclear NICD in undifferentiated MSC after 24 h of stimulus with Mg^2+^. (**d**) Nuclear protein levels of NICD after 24 h of osteogenic stimulus with Mg^2+^ or 2APB. (**e**) NICD expression in MSC and differentiated osteoblast from MSC plus 24 h of Mg^2+^ stimulus after 21 days of treatment.

Confocal microscopy analysis of Notch1 intracellular domain (NICD) showed that Mg^2+^ supplementation produced an increase in nuclear translocation of NICD (Fig. 4a). Osteogenic differentiation decreased the nuclear NICD as compared with UC while Mg^2+^ supplementation increased the presence of nuclear NICD. In OB cells treated with 2-APB the DAPI-NICD co-localization was almost inexistent. As it is observed in the last column of Fig. 4a as compared to 0.8 mM, after Mg addition there was an increase of green pixels (NICD) matching with blue pixels (DAPI), demonstrating nuclear colocalization of NICD protein. Similarly, Fig. 4b shows that a moderate increase of Mg^2+^ in the osteogenic medium upregulated the mRNA expression of HEY2, one of the classic Notch target genes. The opposite effect was observed by the inhibition of the Mg^2+^ transporter TRPM7 with 2-APB, which reduced the nuclear translocation of NICD (Fig. 4a) and Notch activation (Fig. 4b). To examine a likely direct effect of MgCl_2_, the nuclear expression of NICD was evaluated after 24 h of Mg supplementation on MSC or MSC plus osteogenic stimulus. The addition of MgCl_2_ for 24 h increased the nuclear protein expression of NICD as it was confirmed by western blotting (Fig. 4c). However, when MgCl_2_ was administrated for 24 hours in presence of an osteogenic stimulus the levels of nuclear NICD expression were similar to those found with basal Mg^2+^ content (Fig. 4d). The administration of MgCl_2_ for 24 h to differentiated osteoblasts or 2-APB treated cells for 21 days did not modify the nuclear NICD protein expression (Fig. 4e). A high NICD protein expression was also detected by immunoblotting of nuclear protein extracts from MSC and differentiated osteoblasts with Mg^2+^ supplementation after 21 days (Supplementary Figure S1). However, NICD expression was again highly induced in undifferentiated MSC. Taken together, these data suggest that Mg^2+^ activates NICD nuclear translocation on undifferentiated MSC but not in differentiated osteoblasts.

### Magnesium promotes maturation and distribution of osteoblast into decellularized scaffolds

#### Scanning Electron Microscopy (SEM)

**Figure 5:**
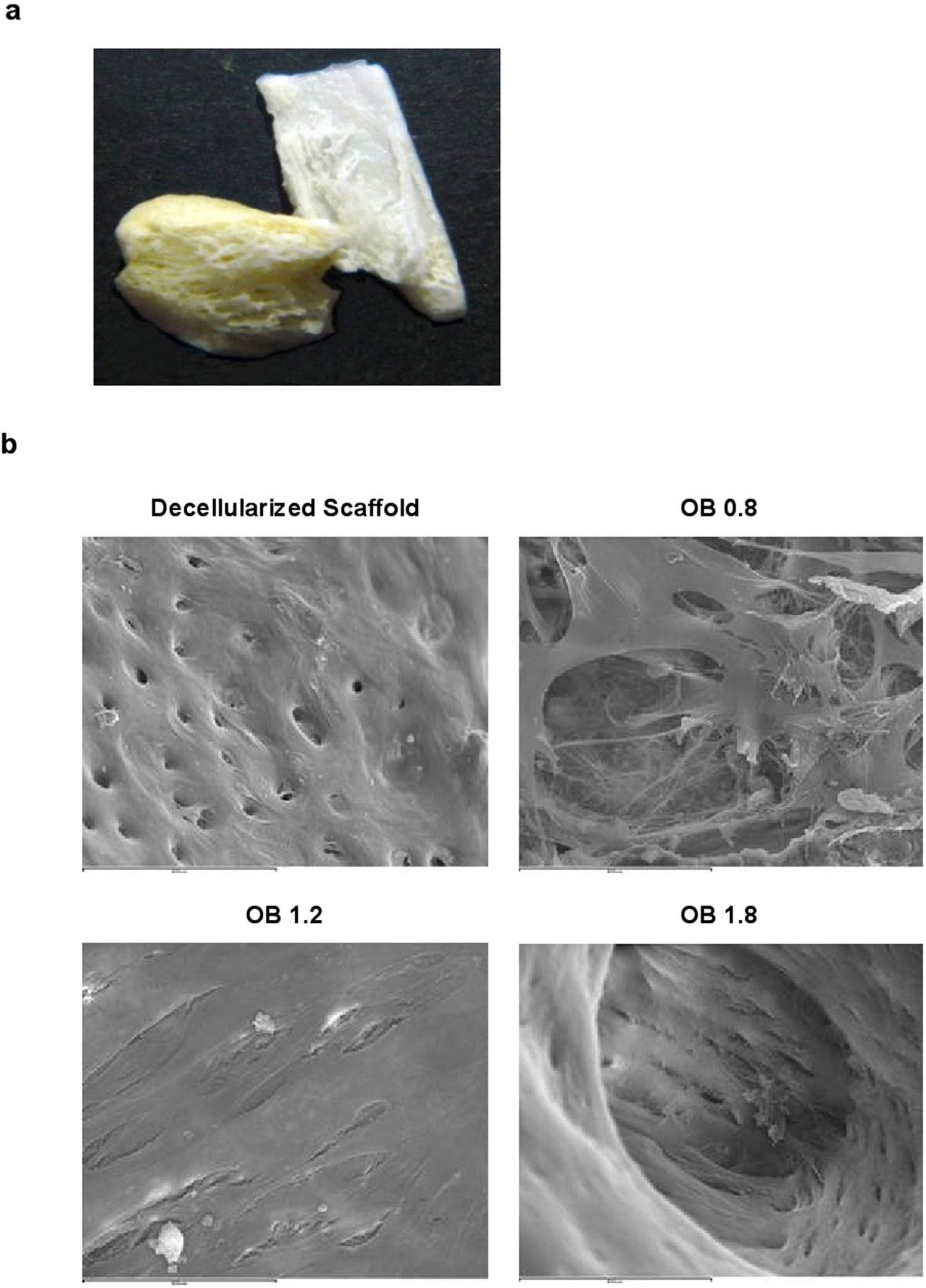
MgCl_2_ supplementation promotes attachment and osteogenesis of MSC on decellularized bone scaffolds. (**a**) Macroscopic picture of rat decellularized bone scaffolds used in this study. Pictures show SEM images of (**b**) decellularized bone scaffold, (**c**) re-cellularizated scaffolds after 21 days of osteogenic differentiation with basal Mg^2+^ (0.8 mM), where spindle-shaped cells attached to the scaffold were observed. Mineralization, cell attachment and proliferation were enhanced dose-dependently as it is showed in (**d**) Mg^2+^ 1.2 mM and (**e**) Mg^2+^ 1.8 mM. Scale bar 800 µm.

In decellularized scaffolds (Fig. 5a), osseous matrix was observed as series of uniform and regular depressions corresponding to ducts osteons (Fig. 5b). Whole bone surface and cavities were covered with a thin membrane with scarce cells. In the scaffolds recellularized with MSC and treated with basal levels of Mg^2+^ (0.8 mM) (Fig. 5c), bone matrix was covered with an irregular membrane with abundant cells following an unspecific distribution and presenting a stellate morphology. After Mg^2+^ supplementation (1.2 mM), MSC formed a uniformly organized layer over the bone surface, of smooth aspect and with abundant cellular proliferation filling the osteon canals (Fig. 5d). In the scaffolds treated with 1.8 mM Mg^2+^, a continuous and regular layer of germinal bone tissue that fully occupied the scaffolds, was observed (Fig. 5e).

#### Transmission Electron Microscopy (TEM)

**Figure 6:**
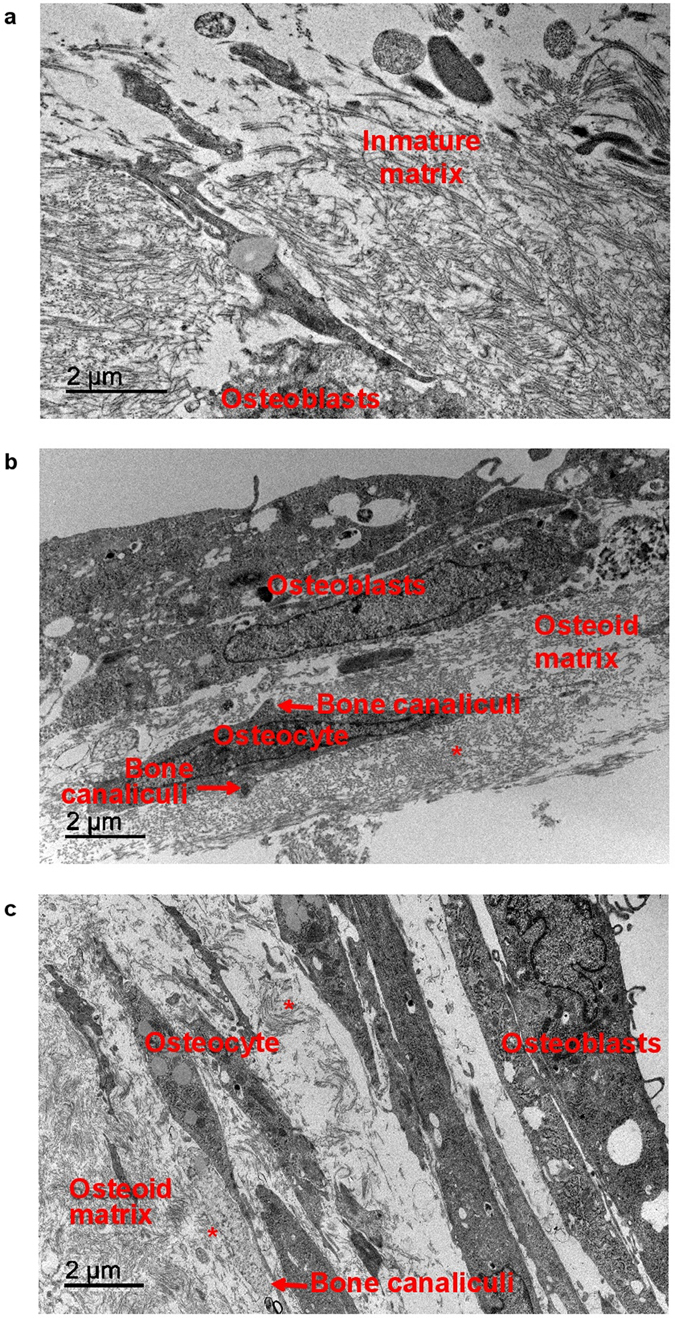
Rat bone marrow MSC differentiated into osteoblasts displayed phenotypic characteristics of osteocyte with MgCl_2_ supplementation. Pictures show TEM images of a MSC after 21 days of osteogenic stimuli with Mg^2+^ 0.8 mM, b 1.2 mM and c 1.8 mM. Scale bar 5 µm. *Collagen fibers; Arrows: Bone Canaliculi.

Cellular layers on decellularized bone scaffolds were analyzed with TEM. During the osteoinduction of MSC with basal Mg^2+^ levels (0.8 mM), collagen-producing blastic cells were poorly organized and with immature matrix (Fig. 6a). After Mg^2+^ supplementation (1.2 mM), the number of osteoblasts increased and they were organized in layers, similar to appositional growth of bone, and more mature osteoblasts (osteocytes) were observed. These osteocytes showed bone canaliculi prolongation and a more organized collagen matrix (Fig. 6b). The highest concentration of MgCl_2_ (1.8 mM) produced a further increase in the number of osteoblast layers (appositional growth), with more mature osteocytes, large bone canaliculi and an organized deposition of collagen (osteoid matrix) (Fig. 6c).

## Discussion

**Figure 7:**
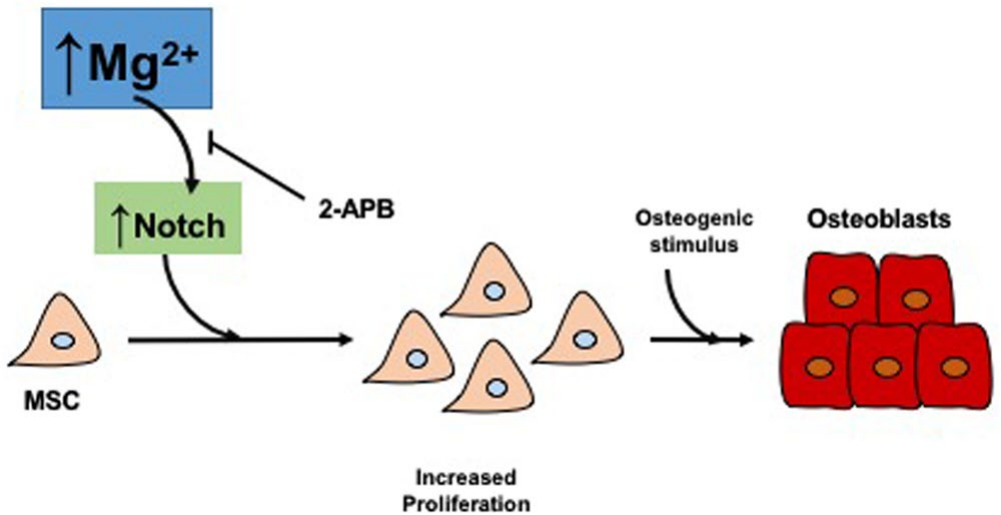
Summary figure. Magnesium supplementation enhances proliferation of MSC. Mechanistically, magnesium ions enter into MSC through TRPM7 channel, increasing Notch Intracellular Domain (NICD) nuclear translocation. Proliferation of MSC contributes to a subsequent osteogenesis. Inhibition of TRPM7 channels by 2-APB decreases the osteogenic potential of magnesium.

In the present study, we have investigated the potential effects of MgCl_2_ in bone marrow MSC during osteogenic differentiation including its effect on anchorage, cellular attachment and differentiation when they are cultured on decellularized bone scaffolds. As it is illustrated in Fig. 7, high concentrations of MgCl_2_ (1.2 mM or 1.8 mM) enhanced proliferation of rat bone marrow MSC in a dose-dependent manner, and increased the subsequent osteogenesis. These effects were not observed after inhibition of the Mg^2+^ channel TRPM7 by 2-APB, which confirmed the key role of intracellular Mg^2+^ in the osteogenesis of MSC. We evaluated the intracellular pathways whereby Mg^2+^ promotes osteogenesis of MSC, finding that MgCl_2_ addition did not increase canonical Wnt/β-catenin pathway activation, although this pathway was activated under osteogenic stimuli. It was interesting to observe that MgCl_2_ supplementation induced the nuclear translocation of NICD and *HEY2* expression. Our results show a direct effect of Mg^2+^ on Notch activation in MSC rather on differentiated osteoblasts from MSC (Fig. 4), suggesting a specific role of Mg^2+^ on the maintenance of stemness of MSC rather on osteogenic process. Furthermore, moderate concentrations of MgCl_2_ considerably promoted osteocyte maturation and enhanced cell attachment to the decellularized bone surface. Note that MgCl_2_ increased the effects of the osteogenic stimuli while conditioned medium with basal levels of Mg^2+^ only produced blastic cells, poorly organized, and without osteocytic phenotype.

The beneficial effects of several Mg^2+^ alloys on bone formation have been widely reported. The balanced combination of Mg^2+^ with different elements such as calcium and phosphate or in Mg-coating prosthesis has demonstrated osteoinductive effects^7, 16^. These effects are supported by the formation of a better structure. The structure and surface characteristic of the biomaterials/scaffolds are key for the attachment and function of the cells and, it may affect the absorption and/or integration of proteins; and in turn, the quality of the anchorage, influences the subsequent cellular responses and tissue regeneration. Minardi *et al*. suggested that Mg^2+^ provides the scaffolds with structural characteristics similar to those of bone, allowing anchorage and proliferation of progenitor cells^9^. Few studies have investigated the active effects of Mg^2+^ on osteogenesis. Recently, Zhang *et al*. have demonstrated that cement formed by a combination of calcium, phosphate and Mg^2+^ increased osteogenesis through a specific interaction between fibronectin and integrin α5β1^17^. Of interest, the authors observed a significant increase in Mg^2+^ concentration (approx. 2.5 mM) after soaking these scaffolds with culture medium; this study support our results demonstrating that Mg^2+^ salts may enhance osteogenesis of progenitor cells. Yoshizawa *et al*. observed that supra-physiological concentrations of Mg_2_SO_4_ (10 mM) also promoted the expression of transcription factors related to *COL10A1* expression^18^. Therefore, these findings suggest that Mg^2+^ salts promote bone formation *in vitro*. In fact, Mg^2+^ is abundant in the skeleton and it is an essential ion in bone development allowing physiological, mineralization and osteogenesis of MSC^19^.

The Mg^2+^ channel TRPM7 is ubiquitously expressed and it is implicated in cellular Mg^2+^ homeostasis^20^. Expression and activity of TRPM7 are modulated by the availability of Mg^2+^ and Ca^2+^. High influx of Mg^2+^ stimulates gene expression of TRPM7 channel and promotes osteoblasts proliferation^21^. Other authors have demonstrated the requirement of TRPM7 for growth and skeletogenesis^22^. The results of these studies are in line with our findings, where TRPM7 inactivation by 2-APB inhibited osteogenesis of bone marrow MSC and significantly reduced Notch1 signaling. The contrary effect was observed with the addition of moderate concentrations of MgCl_2_, which promoted Notch1 signaling activation and increased osteogenesis of the MSC. The central role of this pathway during osteogenesis has been described already^13, 23^, although other works highlights too an inhibition of this pathway during osteogenesis^15^. The interrelationship among Notch1 signaling pathway, Mg^2+^ and osteogenesis is unknown; taken together, our *in vitro* and *in vivo* results suggest that Mg^2+^ supplementation promotes Notch activation tissue-specifically in MSC increasing the pool of osteoprogenitor cells susceptible to be differentiated into osteoblasts in the presence of osteogenic stimuli.

In a recent study, it is demonstrated that the inhibition of Notch pathway in MSC leads to a lesser proliferation and osteogenic capability avoiding fracture healing^24^. Zanotti *et al*. have observed that Notch effects in the skeleton are cell-context-dependent finding different effects on immature osteoblasts or osteocytes^25^. These and other works highlight the potential dimorphic effects of Notch signaling in bone homeostasis^26^.

Finally, and with respect to Mg^2+^ supplementation more studies, considering other important parameters for bone turnover such as PTH or vitamin D, should be led to evaluate with precision the *in vivo* effects of Mg^2+^ supplementation on the mineralization or the osteoid production in the bone.

In addition, we studied the effect of Mg^2+^ on cell organization and osseointegration of progenitor cells on rat decellularized bone. Our results reveal that the supplementation with Mg^2+^ improves cell attachment and increases osteogenic differentiation of MSC cultured on decellularized bone. Transmission electron microscopy analysis showed that Mg^2+^ supplementation increased proliferation and maturation of MSC differentiated into osteocytes with the presence of cytoplasmic prolongations between osteocytes (bone canaliculi) and a well-organized osteoid matrix with a large number of layers similar to the appositional growth observed in bones. Similarly, using scanning electron microscopy analysis, it was shown that Mg^2+^ induced proliferation, differentiation and a more organized distribution of the osteocytes coating the surface of decellularized bone, allowing mineralization and formation of new bone. These data indicate that the bone tissue formed on decellularized rat bone after Mg^2+^ supplementation has a better internal distribution with highly organized structures. Several studies have shown that Mg^2+^ ions may enhance cell attachment and promote bone formation. Moreover, previous studies have demonstrated that Mg^2+^ ions support the initial cell adhesion of MSC and increase proliferation and activation of integrins^27^. Recently, Galli *et al*. also showed that the immersion of threaded screws in a solution of MgCl_2_ (10 mg/ml) leads to enhanced osteogenesis and improves osseointegration^28^.

The data reported in this study could represent a new, feasible and economic strategy to improve bone formation in different types of scaffolds. In addition, these results also suggest that local administration of moderately high levels of Mg^2+^ could be suitable to promote osteogenesis in pathologies in which bone formation is necessary.

This approach would significantly impact the orthopaedic field, and further provides new targets to improve the quality of the materials currently available.

## Methods

### Isolation of rat bone marrow MSC

All experimental protocols were reviewed and approved by the Ethics Committee for Animal Research of IMIBIC in accordance with the ethical guidelines of the Institution and the EC Directive 86/609/EEC for animal experimentation. Twenty male Wistar rats were euthanized by aortic puncture under general anesthesia with pentobarbital sodium (50 mg/kg) and midazolam (4 mg/kg/i.p). Tibiae and femurs were cut at the epiphyses and subsequently perfused with alpha minimal essential medium (αMEM; Sigma-Aldrich CO, St. Louis, MO, USA) containing FBS (15%; Lonza Inc., Walkersville, MD, USA), ultraglutamine (1%; Lonza), penicillin (100 U/mL) and streptomycin (100 µg/mL). Single-cell suspension was generated by filtration through 70 µm cell strainer (BD Biosciences, San Jose, CA, USA). MSC were isolated according to their plastic adherence properties. Following centrifugation and washing with αMEM, bone marrow cells were cultured in 25 cm^2^ flasks (NUNC A/S, Roskilde, DE, USA) with αMEM containing FBS (15%), ultraglutamine (1%), penicillin (100 U/mL), streptomycin (100 mg/mL) and basic fibroblast growth factor (bFGF; 1 ng/mL; PeproTech EC Ltd., London, UK) in a humidified atmosphere with 5% CO_2_ at 37 °C. Fresh α-MEM with FBS (10%), ultraglutamine (1%), penicillin (100 U/mL), streptomycin (100 µg/mL) and bFGF (1 ng/mL) was added after 24 h and changed every 3 days. Once 85–90% confluence was reached, cells were collected using Trypsin-EDTA (Lonza) and seeded in 6-well plates (NUNC) at 13000 cells/cm^2^. Treatments were started as described below when cells reached 90% confluence.

### Osteogenic differentiation of MSC and treatments

MSC were cultured for 21 days in αMEM with FBS (10%), ultraglutamine (1%), penicillin (100 U/mL), streptomycin (100 µg/ml) and osteogenic stimuli based on dexamethasone (1 µM; Sigma-Aldrich), β-glycerol phosphate (10 mM; Sigma-Aldrich) and ascorbic acid (0.2 mM; BAYER, Barcelona, Spain). Basal Mg^2+^ concentration in the medium was 0.8 mM. In order to increase the Mg^2+^ content in the pro-osteogenic medium, MgCl_2_ (Carlo ErbaReagentiSpA, Milano, Italy) was added to achieve final Mg^2+^ concentrations of 1.2 mM and 1.8 mM during the osteogenic stimulus. Fresh medium alone or osteogenic medium supplemented with MgCl_2_ was replaced every 3 days. Furthermore, 2-APB (50 µM; Tocris Bioscience, Bristol, UK) was added to the osteogenic medium containing 0.8 mM of Mg^2+^ during differentiation to determine the effects of inhibiting the Mg^2+^ channel TRPM7. To examine a direct effect of Mg^2+^ supplementation (1.2 and 1.8 m, or 2-APB) for 24 hours on undifferentiated MSC or at the different stages of differentiation (early or mature osteoblasts obtained from MSC) the NICD expression was also analyzed by western blot. All experiments were repeated at least three times.

### RNA isolation and quantitative RT-PCR

Total RNA was extracted with TRI reagent (1 mL; Sigma-Aldrich) and quantified by spectrophotometry (ND-1000, Nanodrop Technologies, Wilmington, DE, USA). cDNA was synthesized from 1 μg of total RNA with a first strand cDNA synthesis kit (Qiagen, Hilden, Germany) in the presence of random hexamers in a final volume of 20 μl at 25 °C for 10 min, followed by 42 °C for 15 min and 95 °C for 3 min. PCR SYBR Green kit (Qiagen N.V., Hilden, Germany) was used to quantify mRNA expression levels. Primers for PCR were synthesized with Oligo software. *RUNX2* (Forward 5′CGG-GAA-TGA-TGA-GAA-CTA-CTC3′ Reverse 5′CGG-TCA-GAG-AAC-AAA-CTA-GGT3′), *OSTERIX* (Forward 5′GTA-CGG-CAA-GGC-TTC-GCA-TCT-GA3′ Reverse 5′TCA-AGT-GGT-CGC-TTC-GGG-TAA-AG3′), *OSTEOCALCIN* (Forward 5′TCT-GAG-TCT-GAC-AAA-GCC-TTC-ATG3′ Reverse 5′TGG-GTA-GGG-GGC-T GG-GGC-TCC3′), and *18-S* (Forward 5′GTA-ACC-CGT-TGA-ACC-CCA-TT3′Reverse 5′CCA-TCC-AAT-CGG-TAG-TAG-CG3′). Primers for *HEY2* (Forward: 5′TCC-AAT-GCT-CAT-AAA-GTC-CGT3′ and Reverse: 5′ TCT-GCA-AAT-GAC-AGT-GGA-TCA3′) were purchased from IDT Integrated DNA Technologies (Leuven, Belgium). The expression level of these genes was evaluated by quantitative RT-PCR (Light cycler, Roche Diagnostics, Basel, Switzerland) with the 2^−ΔΔ^C_t_ method, using ribosomal 18 S RNA as housekeeping control.

### Protein extraction and Western blot analysis

Cytosolic protein was isolated from cells in lysis buffer A, containing Hepes (10 mM), KCl (10 mM), EDTA (0.1 mM), EGTA (0.1 mM), DTT (1 mM), PMSF (0.5 mM), Protease Inhibitor Cocktail (15 µL/mL; Sigma-Aldrich), Igepal CA-630 (0.5%; Sigma-Aldrich), pH 7.9. The suspension was centrifuged, and the supernatant (cytosolic extract) was stored. Nuclear extracts were obtained by incubating the pellet obtained from the cytosolic extract in lysis buffer B, containing HEPES (20 mM), NaCl (0.4 M), EDTA (1 mM), EGTA (1 mM), DTT (1 mM), PMSF (0.5 mM), Protease Inhibitor Cocktail (15 µL/mL), pH 7.9. Protein concentration was determined by Bradford assay (Bio-Rad Laboratories, Hercules, CA, USA). To determine specific protein contents, 20 µg of nuclear cell lysates were analyzed by immunoblotting using antibodies against proliferating cell nuclear antigen (PCNA; 1:100; Santa Cruz Biotechnology INC, Dallas, TX, USA), Cyclin D1 (1:500; Cell Signaling Technology Inc., Danvers, MA, USA) and cleaved Notch 1 (Val1744) (1:200; Cell Signaling Technology) as primary antibodies, and horseradish peroxidase-conjugated goat anti-mouse (1:10000; Santa Cruz Biotechnology) and goat anti-rabbit (1:10000; Santa Cruz Biotechnology) as secondary antibodies. Transcription factor II B (TFIIB; 1:1000; Cell Signaling Technology) was used as loading control.

### Alkaline phosphatase activity quantification

2 µg of cytoplasmic cell lysates were incubated in p-nitrophenol phosphate (2 mM; Sigma-Aldrich) for 30 min at 37 °C. The reaction was stopped by adding NaOH (3 M), and alkaline phosphatase (ALP) activity was measured by quantifying absorption at 405 nm. ALP activity was expressed as µmol of hydrolyzed p-nitrophenol phosphate per min and per mg of protein versus undifferentiated control cells.

### Alizarin red S staining

Matrix mineralization was detected by alizarin red S staining. Cells were washed twice with PBS, fixed with para-formaldehyde (2%) and sucrose (1%) for 15 min and subsequently washed 3 times with PBS. Then, cells were stained with alizarin red S pH 4.1 (40 mM; Sigma-Aldrich) for 20 min, and washed 4 times for 5 min with water at pH 7. Finally, water was removed and samples were dried at room temperature. Plates were scanned in a WIFI OKI Scanner (Madrid, Spain).

### Immunofluorescence analysis

For immunofluorescence analysis cells were cultured upon glass coverslips in 6-well plates. After 14 days of treatment, cells were fixed with cold methanol for 20 min and subsequently washed with PBS. Fixed cells were incubated with the antibodies diluted in BSA (1%; Sigma-Aldrich) in PBS. Primary antibodies against cleaved Notch 1 (Val1744) (1:50) and β-catenin (1:75; BD Biosciences, San Jose, CA, USA) were incubated for 1 h at 4 °C. Subsequently, cells were washed with PBS and incubated with Alexa Fluor 488 anti-mouse (1:500; Invitrogen Ltd., Paisley, UK) diluted in BSA (1%) in PBS. Cell nuclei were visualized with the nuclear dye 4′,6-diamino-2-phenylindole dihydrochloride (DAPI) (Invitrogen). Pictures were obtained at 40X in Axio Observer Z1 Inverted Confocal microscope (LSM5 Exciter Zeiss, Jena, Germany). ImageJ software (National Institutes of Health, Bethesda, MD, USA) was used to analyze the confocal images.

### FGF23 secretion quantification

Supernatants from cultures were collected and pooled, and intact fibroblast growth factor 23 (FGF23) secretion was determined by using a specific ELISA kit (Kainos Laboratories, Tokyo, Japan).

### Rat femurs decellularization and scaffolds preparation

Bone scaffolds were obtained as it has been previously reported by Shahabipour^29^. Briefly, rat femurs and tibiae were cut longitudinally in 5 mm pieces. Subsequently, bone pieces were boiled 4 times for 5 minutes to remove fat tissues. Pieces were stored overnight at −20 °C before decellularization. Bone specimens were thawed at room temperature, washed with PBS and placed in liquid nitrogen for 2 minutes. Then, pieces were maintained in distilled water at room temperature and washed with PBS. The freeze-thaw process was repeated five times to lysate the cells. Bone scaffolds were decellularized in SDS (2.5%) for 24 hours at 37 °C with gentle shaking. Then, bone specimens were washed with PBS twice for 15 minutes to remove SDS, washed in ethanol (70%) and maintained in PBS for 30 minutes with shaking at room temperature.

### Scanning Electron Microscope (SEM)

Decellularized bone fragments were cut in small pieces and washed in buffer solution for 15 minutes to remove debris. Then samples were kept in glutaraldehyde (2.5%). Decellularized bones without addition of MSC were also analyzed to ensure the decellularization process. Bone scaffolds were mounted on the SEM specimen stubs with carbon tape and were carbon-coated. Samples were analyzed and photographed with a Hitachi S520 SEM (Tokyo, Japan).

### Transmission Electron Microscope (TEM)

Bone marrow MSC were cultured in the presence of different concentrations of MgCl_2_ (0.8, 1.2 and 1.8 mM) on rat femur scaffolds. After 21 days of culture, the cellular layer was removed from the surface of the bone scaffolds and they were analyzed by electronic microscopy. For the ultrastructural study, randomly selected samples of decellularized bone scaffolds were primarily fixed in a glutaraldehyde (2%) solution in phosphate buffer (0.1 M) pH 7.4 overnight at 4 °C and then re-fixed in osmium tetroxide (1%) in phosphate buffer (0.1 M) pH 7.4 for 30 minutes. After dehydration in graded ethanol series and embedding in araldite, semi-thin and ultra-thin sections were cut with a LKB ultramicrotome. Ultra-thin sections were viewed and photographed in a Philips CM10 transmission electron microscope.

### Statistical analysis

Differences between means for three or more groups were assessed by T-test. A P-value < 0.05 was considered significant. Statistical analyses were performed with the assistance of GraphPad Prism version 6.1 software (GraphPad Software, Inc., La Jolla, CA, USA).

## Electronic supplementary material

Supplementary Figures S1–S4

## References

[1] Oryan A, Alidadi S, Moshiri A, Maffulli N (2014). Bone regenerative medicine: classic options, novel strategies, and future directions. J. Orthop. Surg. Res..

[2] Garcia-Gareta E, Coathup MJ, Blunn GW (2015). Osteoinduction of bone grafting materials for bone repair and regeneration. Bone.

[3] Bucholz, R. W. Nonallograft osteoconductive bone graft substitutes. *ClinOrthopRelat Res* 44–52 (2002). 10.1097/00003086-200202000-00006 11937865

[4] Giannoudis PV, Dinopoulos H, Tsiridis E (2005). Bone substitutes: an update. Injury.

[5] Silber JS (2003). Donor site morbidity after anterior iliac crest bone harvest for single-level anterior cervical discectomy and fusion. Spine Phila Pa 1976.

[6] Kanazawa I (2007). A case of magnesium deficiency associated with insufficient parathyroid hormone action and severe osteoporosis. Endocr J..

[7] Farraro KF, Kim KE, Woo SL, Flowers JR, McCullough MB (2014). Revolutionizing orthopaedic biomaterials: The potential of biodegradable and bioresorbable magnesium-based materials for functional tissue engineering. J. Biomech..

[8] Kraus T (2012). Magnesium alloys for temporary implants in osteosynthesis: *in vivo* studies of their degradation and interaction with bone. Acta Biomater.

[9] Minardi S (2015). Evaluation of the osteoinductive potential of a bio-inspired scaffold mimicking the osteogenic niche for bone augmentation. Biomaterials.

[10] Witte F (2005). *In vivo* corrosion of four magnesium alloys and the associated bone response. Biomaterials.

[11] Han P (2015). *In vitro* and *in vivo* studies on the degradation of high-purity Mg (99.99wt.%) screw with femoral intracondylar fractured rabbit model. Biomaterials.

[12] Abdallah BM, Jafari A, Zaher W, Qiu W, Kassem M (2015). Skeletal (stromal) stem cells: an update on intracellular signaling pathways controlling osteoblast differentiation. Bone.

[13] Lin G, Hankenson KD (2011). Integration of BMP, Wnt, and Notch signaling pathways in osteoblast differentiation. J. Cell. Biochem..

[14] Engin F, Lee B (2010). NOTCHing the bone: insights into multi-functionality. Bone.

[15] Hilton MJ (2008). Notch signaling maintains bone marrow mesenchymal progenitors by suppressing osteoblast differentiation. Nat. Med..

[16] Staiger MP, Pietak AM, Huadmai J, Dias G (2006). Magnesium and its alloys as orthopedic biomaterials: a review. Biomaterials.

[17] Zhang J (2015). Magnesium modification of a calcium phosphate cement alters bone marrow stromal cell behavior via an integrin-mediated mechanism. Biomaterials.

[18] Yoshizawa S, Brown A, Barchowsky A, Sfeir C (2014). Magnesium ion stimulation of bone marrow stromal cells enhances osteogenic activity, simulating the effect of magnesium alloy degradation. Acta Biomater.

[19] Rude RK, Gruber HE (2004). Magnesium deficiency and osteoporosis: animal and human observations. J.Nutr. Biochem..

[20] Hoenderop JG, Bindels RJ (2005). Epithelial Ca2+ and Mg2+ channels in health and disease. J. Am. Soc. Nephrol..

[21] Abed E, Moreau R (2009). Importance of melastatin-like transient receptor potential 7 and magnesium in the stimulation of osteoblast proliferation and migration by platelet-derived growth factor. AmJPhysiol Cell Physiol.

[22] Elizondo MR (2005). Defective skeletogenesis with kidney stone formation in dwarf zebrafish mutant for trpm7. Curr. Biol..

[23] Long F (2012). Building strong bones: molecular regulation of the osteoblast lineage. Nat. Rev. Mol. Cell. Biol.

[24] Wang C (2016). NOTCH signaling in skeletal progenitors is critical for fracture repair. J. Clin. Invest..

[25] Zanotti S (2008). Notch inhibits osteoblast differentiation and causes osteopenia. Endocrinology.

[26] Engin F (2008). Dimorphic effects of Notch signaling in bone homeostasis. Nat. Med.

[27] Kim BS, Kim JS, Park YM, Choi BY, Lee J (2013). Mg ion implantation on SLA-treated titanium surface and its effects on the behavior of mesenchymal stem cell. Mater. Sci. Eng. CMater. Biol. Appl.

[28] Galli S (2014). Local release of magnesium from mesoporous TiO2 coatings stimulates the peri-implant expression of osteogenic markers and improves osteoconductivity *in vivo*. Acta Biomater.

[29] Shahabipour F, Mahdavi-Shahri N, Matin MM, Tavassoli A, Zebarjad SM (2013). Scaffolds derived from cancellous bovine bone support mesenchymal stem cells’ maintenance and growth. Vitro Cell DevBiolAnim.

