# Supplementary material for: Magnesium Chloride promotes Osteogenesis through Notch signaling activation and expansion of Mesenchymal Stem Cells
Source: Sci Rep. 2017 Aug 10;7:7839. doi: 10.1038/s41598-017-08379-y (PMC5552799; doi:10.1038/s41598-017-08379-y)
Supplement: Supplementary file 1 — Supplementary Figures S1–S4 [file 41598_2017_8379_MOESM1_ESM.pdf]

Magnesium Chloride promotes Osteogenesis through Notch signaling activation and expansion of Mesenchymal Stem Cells

Juan M. Díaz-Tocados[1,2,3,4#], Carmen Herencia[1,2,3,#], Julio M. Martínez-Moreno[1,2,3], Addy Montes de Oca[1,2,3], Maria E. Rodríguez-Ortiz[4,5], Noemi Vergara[1,2,3], Alfonso Blanco[6], Sonja Steppan[7], Yolanda Almadén [1,8,3,9,*], Mariano Rodríguez[1,2,3,4,+], Juan R. Muñoz-Castañeda[1,2,3,4,+].

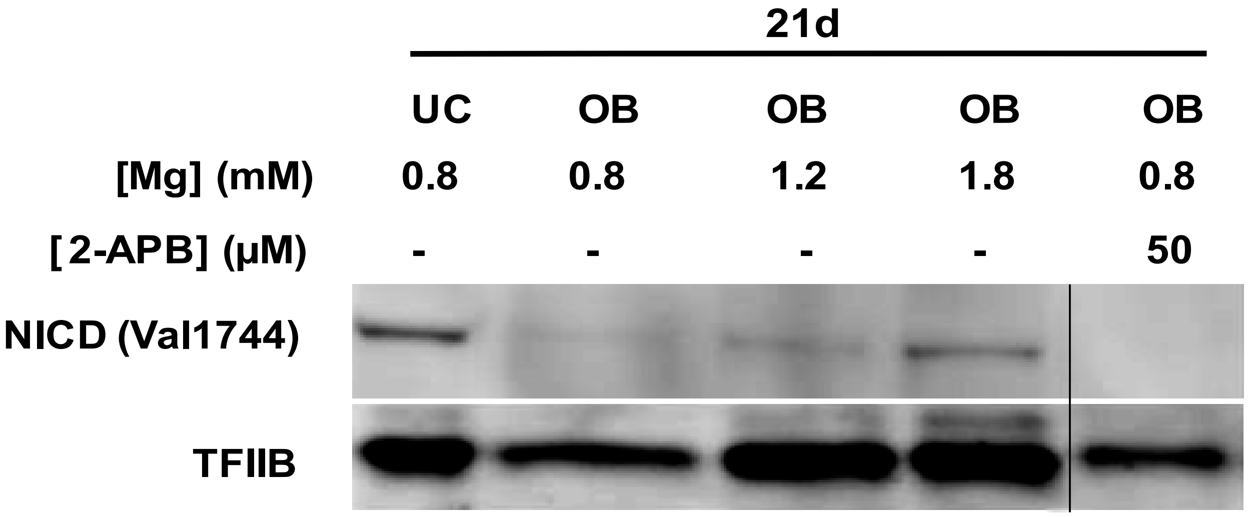

Supplementary Figure S1. Nuclear NICD levels after 21 days in MSC, differentiated osteoblasts with basal Mg^{2+} (0.8 mM), Mg^{2+} supplementation (1.2 or 1.8 mM) or TRPM7 inhibition (2-APB). Line separates results from different part of the gel using same exposure and protein load. UC - undifferentiated cells, OB - osteoblasts. Bars show mean ± SEM. Original magnification: 400x.

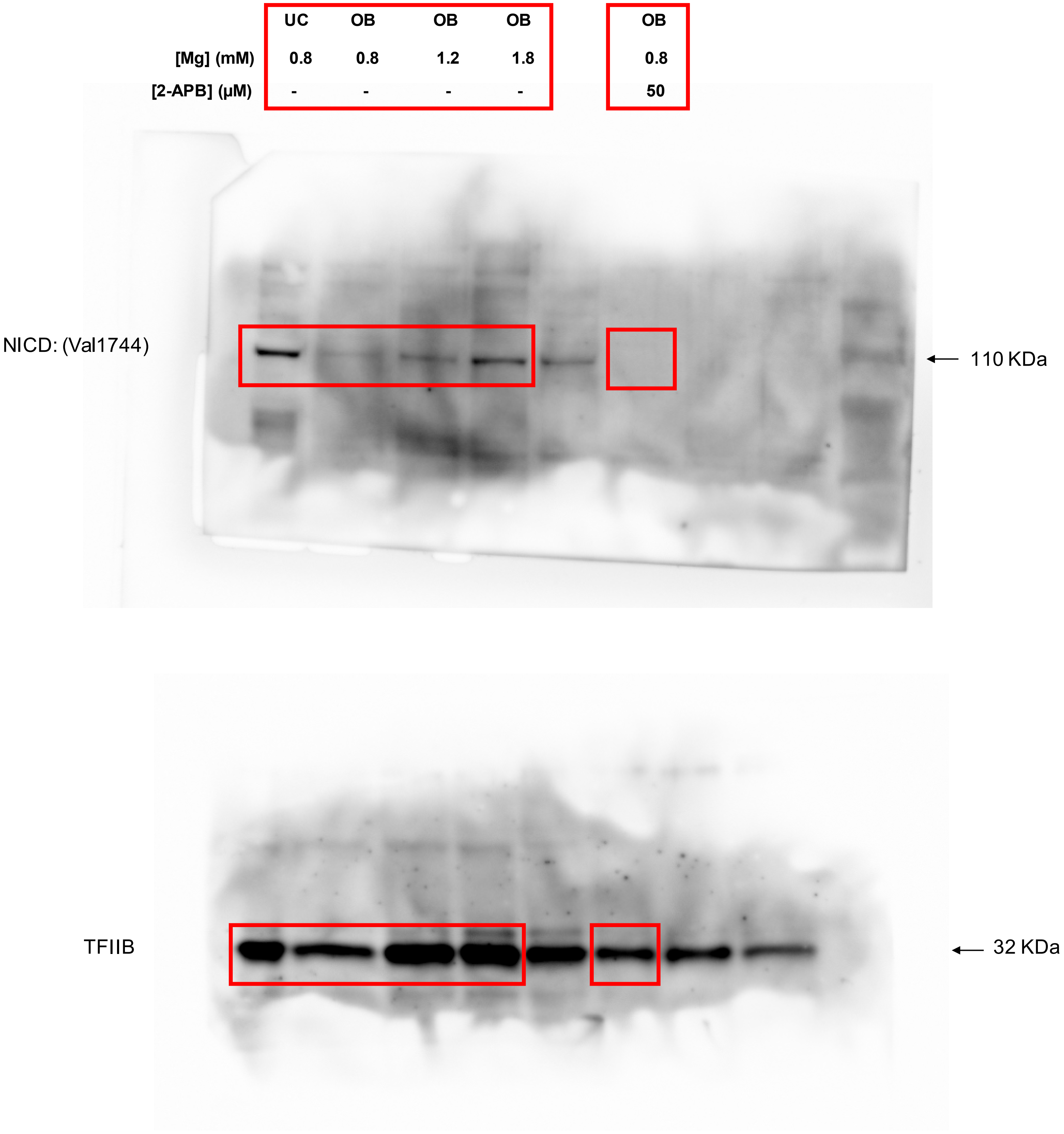

Supplementary Figure S2. Full-length gels corresponding to figure S1. Red boxes indicate lanes showed in the figure S1.

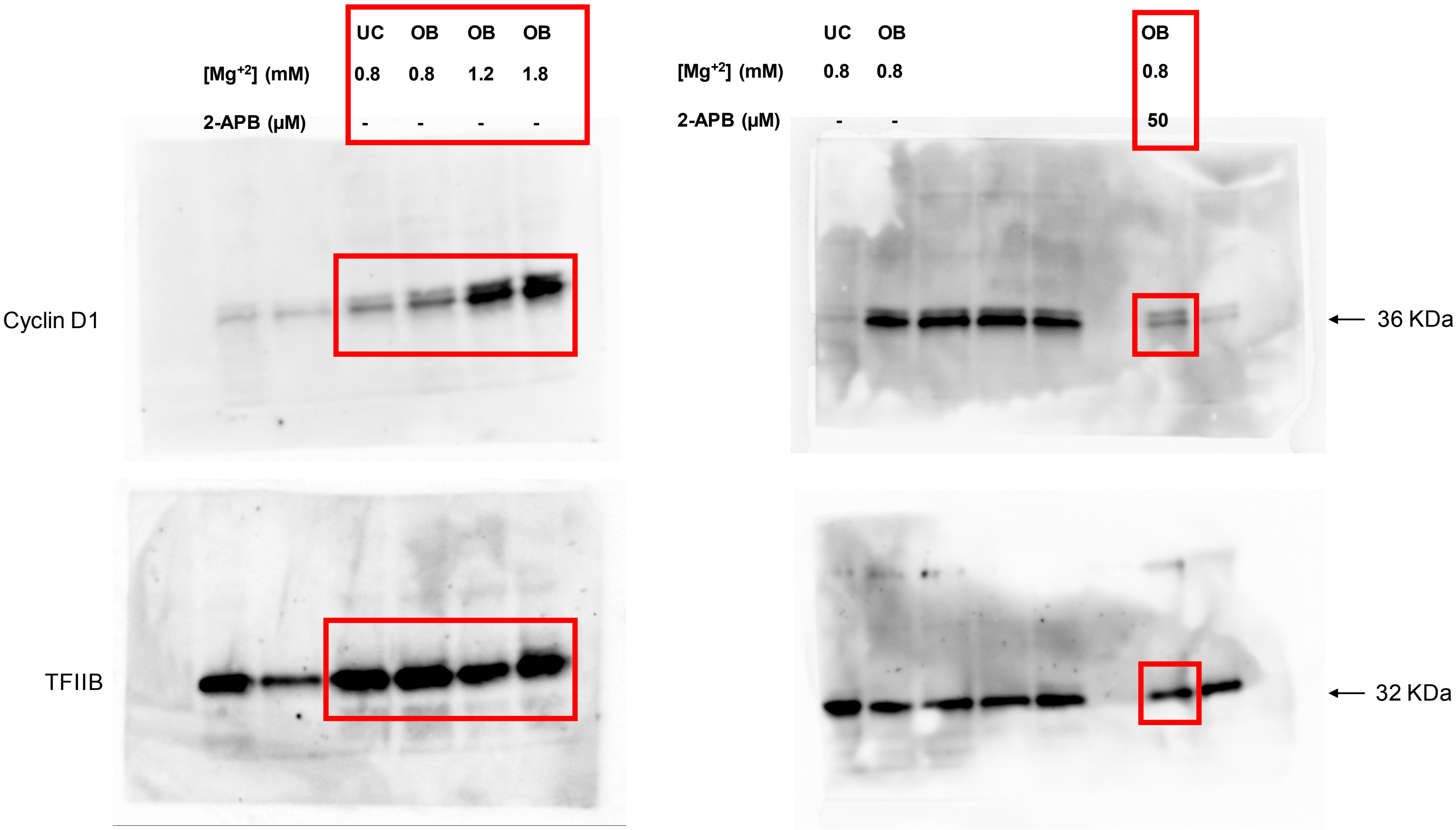

Supplementary Figure S3. Full-length gels corresponding to figure 2g. Red boxes indicate lanes showed in the figure 2g.

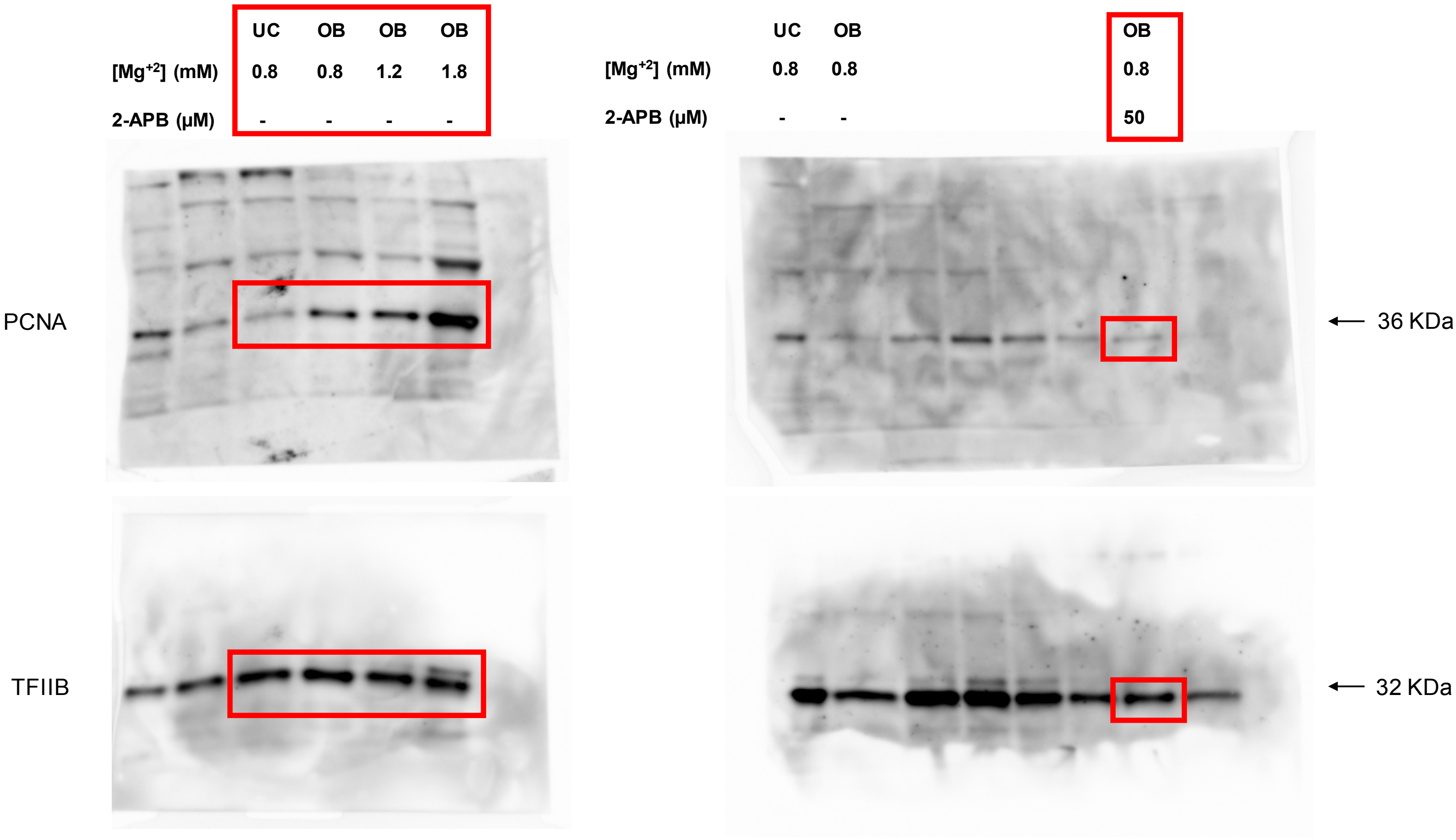

Supplementary Figure S4. Full-length gels corresponding to figure 2h. Red boxes indicate lanes showed in the figure 2h.
